# Embryonic Hyperglycemia Delays the Development of Retinal Synapses in a Zebrafish Model

**DOI:** 10.3390/ijms23179693

**Published:** 2022-08-26

**Authors:** Abhishek P. Shrestha, Ambalavanan Saravanakumar, Bridget Konadu, Saivikram Madireddy, Yann Gibert, Thirumalini Vaithianathan

**Affiliations:** 1 Department of Pharmacology, Addiction Science, and Toxicology, College of Medicine, University of Tennessee Health Science Center, Memphis, TN 38163, USA; 2 Program in Biology, Rhodes College, Memphis, TN 38112, USA; 3 Department of Cell and Molecular Biology, University of Mississippi Medical Center, Jackson, MS 39216, USA; 4 Department of Ophthalmology, Hamilton Eye Institute, University of Tennessee Health Science Center, Memphis, TN 38163, USA

**Keywords:** ribbon synapse, zebrafish (*Danio rerio*) model, retinal development, *ribeye* protein, hyperglycemia, synaptic proteins

## Abstract

Embryonic hyperglycemia negatively impacts retinal development, leading to abnormal visual behavior, altered timing of retinal progenitor differentiation, decreased numbers of retinal ganglion cells and Müller glia, and vascular leakage. Because synaptic disorganization is a prominent feature of many neurological diseases, the goal of the current work was to study the potential impact of hyperglycemia on retinal ribbon synapses during embryonic development. Our approach utilized reverse transcription quantitative PCR (RT-qPCR) and immunofluorescence labeling to compare the transcription of synaptic proteins and their localization in hyperglycemic zebrafish embryos, respectively. Our data revealed that the maturity of synaptic ribbons was compromised in hyperglycemic zebrafish larvae, where altered *ribeye* expression coincided with the delay in establishing retinal ribbon synapses and an increase in the immature synaptic ribbons. Our results suggested that embryonic hyperglycemia disrupts retinal synapses by altering the development of the synaptic ribbon, which can lead to visual defects. Future studies using zebrafish models of hyperglycemia will allow us to study the underlying mechanisms of retinal synapse development.

## 1. Introduction

The metabolic disorder diabetes mellitus (DM) is characterized by chronic elevation of blood glucose caused by inadequate production of insulin or the inability to utilize the insulin that is produced. One of the most common severe complications of long-term hyperglycemia is diabetic retinopathy, which can lead to vision loss. Chronic hyperglycemia incites the expression of proinflammatory mediators that can break down the blood–retinal barrier, resulting in fragile, leaky blood vessels, hemorrhage, neuronal cell death, and vision loss [1,2,3]. Interestingly, retinal thickness is significantly reduced in children from pregnancies of diabetic women relative to those of nondiabetic women, suggesting that prenatal diabetes is likely to be associated with changes in retinal morphology in the offspring [4]. Consequently, there is a critical need to determine the effects of hyperglycemia in an animal model, where the developing eye is accessible and amenable to manipulation.

The visual system of the zebrafish (*Danio rerio*) is fundamentally similar to that of humans [5], with nearly 70% of the relevant zebrafish genes being similar to their human orthologs [6]. The availability of numerous hyperglycemic zebrafish models facilitates the use of both genetic and non-genetic approaches to study DM in the eye, including the effects of hyperglycemia and the development of diabetic retinopathy. The aquatic zebrafish environment can be manipulated to study the specific effects of a chemical agent on embryo development, which is external, rapid, and easily visualized. For example, immersion of zebrafish in water containing high glucose results in the developmental changes of hyperglycemia [7,8], thereby facilitating our study of these changes. Overall, their relatively easy maintenance, high fecundity, small size, and manipulatable environment make zebrafish an excellent model for vertebrate developmental biology.

Two recent studies reported abnormal retinal development in hyperglycemic zebrafish embryos [7,8]. Although altered retinal thickness corresponds to decreased numbers of photoreceptors, horizontal cells, and ganglion cells in hyperglycemic zebrafish embryos, it remains unclear whether these embryos exhibit any other deficits in the establishment of retinal synapses that would result in the observed visual defects [7,8]. The fundamental process of developmental synaptogenesis builds neural circuits in the brain. Synapses are constantly formed and eliminated during development to ensure correct synaptic connectivity between neurons—in this case, for the development of normal visual function. The retinas of five-day-old zebrafish larvae already possess visual functionality and exhibit mature anatomical features, comprising three nuclear layers and two plexiform layers. The outer nuclear layer (ONL) contains the photoreceptor cell bodies; the inner nuclear layer (INL) contains the bipolar, amacrine, horizontal, and Müller glial cell bodies; and the ganglion cell layer (GCL) contains the ganglion cell bodies. The synapses connecting the photoreceptors and bipolar cells with horizontal cells occupy the outer plexiform layer (OPL), while the synapses connecting the ganglion cells with bipolar cells and amacrine cells reside in the inner plexiform layer (IPL). 

In this study, we primarily focused on the development of the OPL because any changes in the establishment of this primary synapse can affect the entire visual pathway, and previous studies found a loss of neurons that establish the OPL synapse in hyperglycemic zebrafish embryos. In the zebrafish, the cone mosaic presence in the ventral retina is found 54 h post-fertilization (hpf) [9], and the rod terminals are visible as early as 5 dpf [10]. Interestingly, recent studies, using both genetic and nutritional models of embryonic hyperglycemia, found that rod and cone photoreceptor cells are significantly decreased [8]. Thus, any changes we report in the OPL synapse development in the current studies may apply to rod and cone photoreceptors similarly. Photoreceptors establish unique ribbon synapses with second-order neurons in the OPL. The main structural component of synaptic ribbons is the *ribeye* protein. It has been hypothesized that specific functions are executed by docking *ribeye*-specific proteins to the scaffold of the synaptic ribbon. In the retina, synaptic ribbons are also found in the terminals of bipolar cells in the IPL. The structure of the *ribeye* protein consists of a unique N-terminal domain (A) and a C-terminal domain (B) that is identical to C-terminal binding protein 2 (CtBP2) [11,12,13]. Zebrafish have two copies of the *ribeye* gene, *ribeye a* and *ribeye b*, which share 54% sequence similarity in the N-terminal A domain and 91% sequence similarity in the C-terminal B/CtBP2 domain. *Ribeye a* is expressed in both photoreceptors and bipolar cells, while *ribeye b* is detected only in photoreceptors [14].

Here, we examined the expression patterns of synaptic proteins that establish the OPL using quantitative RT-PCR (RT-qPCR) and immunohistochemistry (IHC) in hyperglycemic zebrafish larvae. Our data revealed that after 24 h of exposure to glucose in embryonic media, the whole body and retina of zebrafish embryos exhibited a dramatic increase in free glucose levels. At this time point, we detected significantly reduced levels of *ribeye b* transcripts in hyperglycemia embryos compared to control embryos. At 120 h post-fertilization (hpf), we found that the number and size of synaptic ribbons in the photoreceptors were significantly reduced, as was accumulation of the L-type voltage-gated calcium channel protein Ca_v_1.4/cacna1f in the photoreceptor synapse on zebrafish larvae exposed to high glucose on alternating days. Co-immunolabeling of postsynaptic proteins, including membrane-associated guanylate kinase (MAGUK) and glutamate receptor 2 (GluR2/3), demonstrated loss of ribbon synapses in the OPL. However, specific labeling with *ribeye a* protein demonstrated that the IPL synaptic ribbons were largely preserved. Indeed, specific labeling of protein kinase C α (PKCα) revealed that the terminals and soma of rod bipolar cells (RBCs) were similar in the control and hyperglycemic zebrafish embryos. Thus, we report for the first time that the expression of *ribeye* transcripts and proteins and the morphology of ribbon synapses were altered in the hyperglycemic embryonic zebrafish model.

## 2. Results

**Figure 1:**
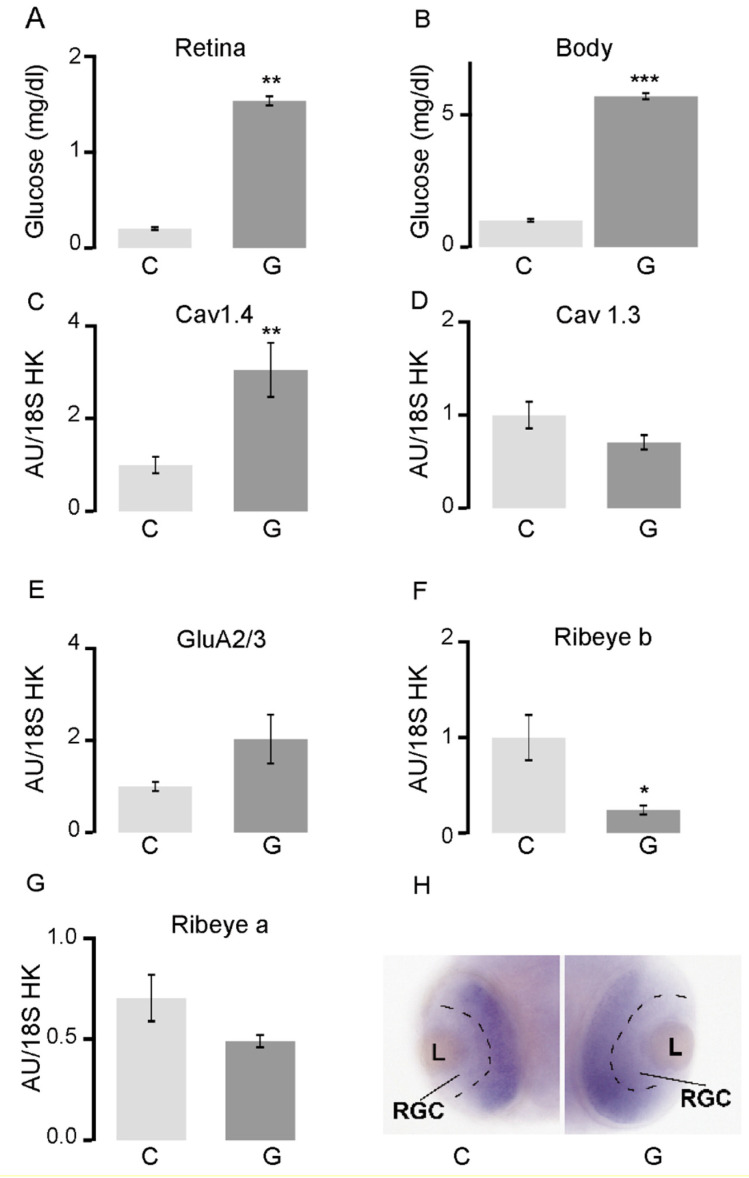
In zebrafish larvae, hyperglycemia results in altered transcription of retinal synaptic genes and localization of synaptic proteins. (**A**) Glucose concentration at 48 hpf in the retina and (**B**) in the rest of the body in embryos exposed to 4% glucose 24–48 hpf; (**C**–**G**) RT-qPCR analysis of the whole retina in embryos exposed to 4% glucose 24–48 hpf. All transcript levels were normalized to those of 18S rRNA. The levels of *ca_v_1.4a* transcripts increased, those of *ca_v_1.3a*, *GluR2/3*, and *ribeye a* remained unchanged, while those of *ribeye b* decreased in retinas exposed to glucose, relative to controls; (**H**) In situ hybridization for *ribeye a* in the transverse sections in the center of the eye 48 hpf in control embryos (**C**) and those exposed to 4% glucose (**G**). The separation between the already defined retinal ganglia cell layer and the developing remainder of the retinal layer is marked by a black dashed line. * *p* < 0.05; ** *p* < 0.01; *** *p* < 0.001. Abbreviations: C, control; G, exposed to glucose; GluR2/3, glutamate receptor; hpf, hours post-fertilization; rRNA, ribosomal RNA; RT-qPCR, reverse transcription quantitative PCR; L, lens; RGC, retinal ganglion cells.

We began to investigate the hyperglycemic effects on retinal synapses by confirming whether our glucose exposure protocol led to increased glucose levels specifically in the retina of zebrafish embryos. Control embryos were solely exposed to E3 embryonic medium, while those in the glucose treatment group were exposed to 4% glucose from 24 to 48 hpf. At 48 hpf, the embryos were collected, and both retinas were removed from the rest of the embryo’s body. Free glucose analysis revealed that the retinas of zebrafish embryos exposed to glucose possessed a glucose concentration that was increased six times over that of control embryos (Figure 1A), with exposed embryos containing an average of 1.5 mg/dL glucose. We also observed an increased level of glucose in the rest of the body of embryos exposed to glucose, although the level was higher in the body (5 mg/dL) than in the retinas (Figure 1B). These results confirmed the efficacy of our protocol in increasing glucose levels in the embryonic zebrafish retina.

Next, we examined whether this increase in retinal glucose during early developmental stages had any impact on the expression or accumulation of synaptic proteins. Synaptic ribbons are first identified in the ventronasal patch at 65 hpf and by 72 hpf are present in most retinal regions. Many of the first precursors to synaptic ribbons are small round precursor bodies (srPBs) found at 72 hpf that remain in the cytoplasm, unassociated with the postsynaptic elements and perhaps not yet functional [15,16]. By 75 hpf, the srBPs have formed fully mature synaptic ribbons that are functional in all the terminals in the central retina [17]. The *ribeye* protein is the main component of synaptic ribbons and the only known protein unique to this structure [18]. *Ribeye* is formed from an alternative start site of the transcriptional co-repressor CtBP2. Zebrafish have two *ribeye* genes, *ribeye a* and *ribeye b*. While *ribeye a* is expressed in both photoreceptors and bipolar cells, *ribeye b* is only detected in photoreceptors [14].

To reveal whether the increased levels of glucose detected in the retina at 48 hpf (Figure 1A) could affect *ribeye* gene expression, we performed RT-qPCR on whole retinas from each of the control and glucose-exposed embryos. We observed increased levels of L-type voltage-dependent calcium channel (L-VDCC) Ca_v_1.4 transcripts (Figure 1C) but not L-VDCC Ca_v_1.3 transcripts (Figure 1D) in the retina of hyperglycemia larvae, relative to controls. Transcript levels of GluR2, a postsynaptic protein that establishes primary retinal synapses, also exhibited a trend toward upregulation, but the difference was not statistically significant at 48 hpf (Figure 1E). Importantly, we found that the transcript levels of *ribeye b* were significantly reduced in the retina of glucose-exposed embryos, relative to control embryos (Figure 1F). Although the accumulation of *ribeye a* transcripts also exhibited a trend toward reduction in the retinas of hyperglycemic embryos, these changes were not statistically significant at 48hpf (Figure 1G). Since synaptic ribbons can be visualized at 72 hpf, we performed in situ hybridization to reveal the location of *ribeye* expression at 48 hpf and found that *ribeye a* expression was excluded from the retinal ganglion cells (RGC) but was expressed in the remaining cell layers (Figure 1H).

Previous studies demonstrated abnormal retinal development in hyperglycemic zebrafish embryos [7,8]. While the decreased number of photoreceptors, horizontal cells, and ganglion cells corresponded to altered retinal thickness in these embryos, it remained unclear whether the reduced number of retinal cells alone accounted for the observed visual defects or whether there existed any defects in the development of retinal synapses in this model. As noted above, the morphology of the visually responsive retina in 5 dpf zebrafish larvae is anatomically and functionally similar to the retinas of humans and adult zebrafish in its composition of three nuclear layers (ONL, INL, and GCL) separated by two plexiform layers (OPL and IPL). The formation of synapses between the first-order nuclear layer neurons occurs in the plexiform layers. At 5 dpf, the retinal ribbon synapses reach morphological maturity, and the synapses between photoreceptors and second-order neurons mature and become functional for vertical synaptic transmission [15]. Photoreceptor cells and bipolar cells form specialized structures at their presynaptic termini that facilitate their response and adaptation to a wide range of light intensities. Several key components required for the synaptic ribbon structure have been identified [12,18,19], and the complex interactions between these proteins during ribbon synaptogenesis are beginning to be characterized [17,19,20,21,22]. However, it remains unknown whether hyperglycemia can affect the synaptic ribbon complex. While one study shows loss of synaptic ribbons and synaptic connectivity in type-1 diabetic Ins2Akita transgenic mice, the effects of hyperglycemia in retinal ribbon development have not been previously explored [23].

**Figure 2:**
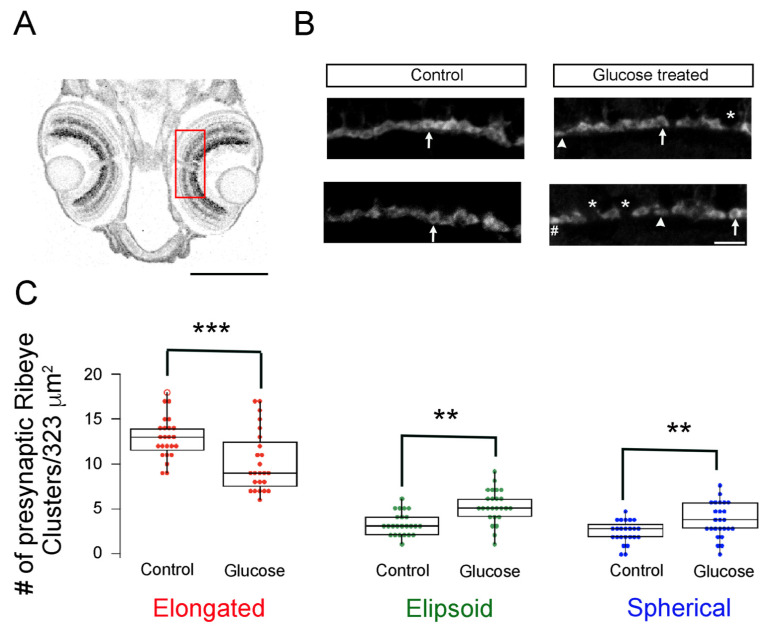
The maturation of synaptic ribbons is delayed in hyperglycemic zebrafish larvae. (**A**) Imaging region of the retina is illustrated with a ROI (red) placed on the transverse section of 5 dpf larvae. Scale bar, 200 μm; (**B**) Maximal intensity projection of confocal z-stacks of retinal sections from control (**left panels**) and hyperglycemic larvae (**right panels**) immunostained with fluorescently labeled antibodies specific for ribeye/CtBP2. Mature (elongated, arrow) and immature (ellipsoid, arrowhead or spherical, #) ribbon morphologies are indicated. * Denotes the gaps between ribbons. Scale bar, 5 μm; (**C**) Quantitative analyses of ribbon morphologies in retinal sections stained for ribeye/CtBP2 were categorized according to morphology, as described in Materials and Methods: mature (elongated, red) and immature (ellipsoid, green or spherical, blue) morphologies were noted and are shown as box-and-whisker plots. Boxes show all values, while whiskers indicate minimum and maximum values. Data are presented as mean values ± SEM. The total number of profiles examined was 521 control larvae and 388 glucose-treated larvae in groups of 50 larvae; the retinas from each of the larvae were processed independently. ** *p* < 0.01; *** *p* < 0.001. Abbreviations: CtBP2, C-terminal binding protein 2; dpf, days post-fertilization; ROI, region of interest; SEM, standard error of the mean.

Therefore, we examined the effects of glucose exposure on the development of the embryonic retinal synapse. We performed a series of IHC assays and confocal imaging to look for possible alterations in retinal synapses and neuronal morphologies from the coronal retinal section of control and hyperglycemic larvae 5 dpf (Figure 2A). All IHC analysis was performed on zebrafish larvae exposed to high glucose on alternating days. We used antibodies specific for the CtBP domain that primarily binds to *ribeye a and b* proteins [24,25] to reveal the morphology and maturity of synaptic ribbons in 5 dpf control and hyperglycemic larvae. CtBP2 proteins were primarily expressed in the OPL of both control and hyperglycemic larvae (Figure 2B). In 5 dpf control larvae, fluorescent labeling of CtBP2 revealed that most photoreceptor ribbons exhibited the well-known horseshoe-shaped morphology, thereby confirming the previous observation that synaptic ribbons are mature at this stage in development. In hyperglycemic larvae, CtBP2 labeling remained unchanged and was comparable to that in control larvae. However, when all the ribbon synapses in the control retinas had adopted the expected elongated and horseshoe-shaped morphologies by 5 dpf, a significant number of *ribeye*-containing structures in the hyperglycemic larvae remained small and round, while a reduced number of these structures resembled mature ribbons (Figure 2B). To quantify the number of photoreceptor ribbon synaptic sites, we measured the length and width of the fluorescent structures stained with anti-CtBP2 antibodies in 323 μm^2^ areas of the OPL of control and hyperglycemic larvae and counted the number of different shapes that materialized (Figure 2C), as described in Materials and Methods. The number of horseshoe-shaped synaptic ribbons stained for CtBP2 significantly decreased in hyperglycemic larvae (Figure 2C, red; *p* < 0.001), while the number of ellipsoids and spherical ribbons significantly increased (Figure 2C, green and blue; *p* < 0.01). We also noted more frequent gaps between CtBP2 clusters in the hyperglycemic larvae, which might represent areas of immature synapse or loss of photoreceptor from the mature synapse. These data suggest that in 5 dpf hyperglycemic larvae, the synaptic sites were not completely absent but had not (yet) matured to the extent observed in control larvae of the same developmental age.

**Figure 3:**
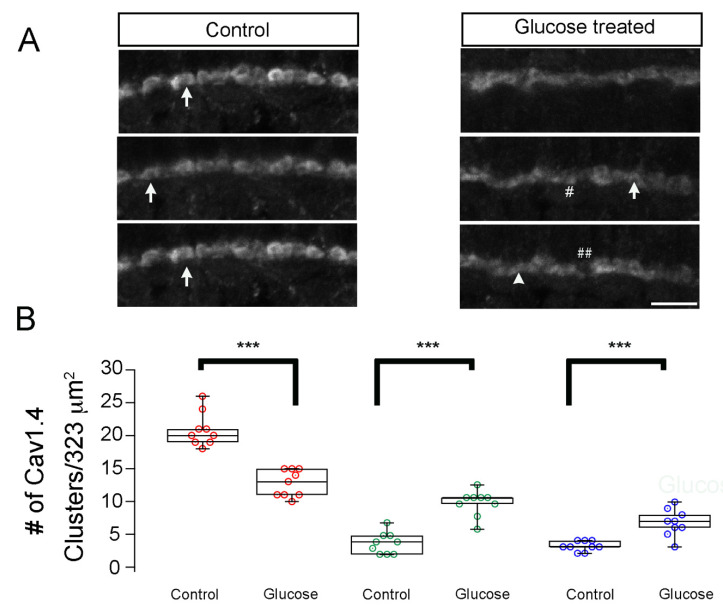
Hyperglycemia leads to developmental changes in Cav1.4/cacna1f accumulation in the OPL of zebrafish larvae. (**A**) Transverse retinal sections from 5 dpf control (**left**) and hyperglycemic (**right**) larvae immunostained with fluorescently labeled antibodies specific for Cav1.4/cacna1f. Maximal intensity projections are shown. Mature (elongated, arrow) and immature (ellipsoid, closed arrows or spherical, #) ribbon morphologies are indicated. ## Denotes the gaps between Cav1.4 proteins. Scale bar, 5 µm. (**B**) Quantitative analyses of IHC for Cav1.4/cacna1f were performed similarly for ribeye/CtBP2 and were categorized according to morphology, as described in Materials and Methods: mature (elongated, red) and immature (ellipsoid, green or spherical, blue) morphologies were noted and are shown as box-and-whisker plots. Boxes show all values, while whiskers indicate minimum and maximum values. Data are presented as mean values ± SEM. *** *p* < 0.001. Abbreviations: Cav1.4/cacna1f, voltage-dependent calcium channel; dpf, days post-fertilization; IHC, immunohistochemistry; SEM, standard error of the mean.

Synaptic ribbon formation could also be regulated by calcium influx via L-VDCCs. In the Ca_v_1.4/cacna1f voltage-dependent calcium channel, both loss- and gain-of-function mutations can impair photoreceptor ribbon development in the mouse retina [26]. Zebrafish possess two Ca_v_1.4/cacna1f genes that are both expressed in the retina, but their transcripts are expressed in a mutually exclusive pattern. Cacna1fa transcripts are expressed in the outer retina, while those of cacna1fb are expressed only in the inner retina, which may suggest their expression in photoreceptor and inner retina neurons, respectively [27]. If the abnormal synaptic ribbons we observed in hyperglycemic larvae resulted from altered Ca_v_1.4/cacna1f properties, we would expect to find altered expression of Ca_v_1.4/cacna1f in 5 dpf hyperglycemic larvae. We, therefore, immunostained the retinas from 5 dpf control and hyperglycemic larvae with fluorescently labeled antibodies specific for Ca_v_1.4/cacna1f [28] and used confocal microscopy to examine its localization. We observed Ca_v_1.4/cacna1f labeling primarily in the retinal OPL (Figure 3A) and not in the IPL, suggesting that Ca_v_1.4/cacna1f localization may be specific to the OPL. Its localization was similar to that of CtBP2, consistent with previous studies [26,27,28,29].

Due to the similar immunosignals of Ca_v_1.4/cacna1f and CtBP2 in photoreceptors, we asked whether differences in the size of the synaptic ribbon were echoed by the pattern of Ca_v_1.4/cacna1f expression. We measured the length and width of Ca_v_1.4/cacna1f cluster, as described in Materials and Methods. As we observed for CtBP2, the number of horseshoe-shaped structures immunostained with antibodies specific for Ca_v_1.4/cacna1f was significantly decreased in the hyperglycemic larvae (Figure 3B red; *p* < 0.001), while the number of ellipsoids and spherical significantly increased (Figure 3B green and blue; *p* < 0.001). These data suggest that the different sizes of ribbons may be linked to the size of the Ca_v_1.4/cacna1f-expressing regions, which would lend support to the previous finding of tight coupling between ribbon length and the length of the active zone [26,29].

**Figure 4:**
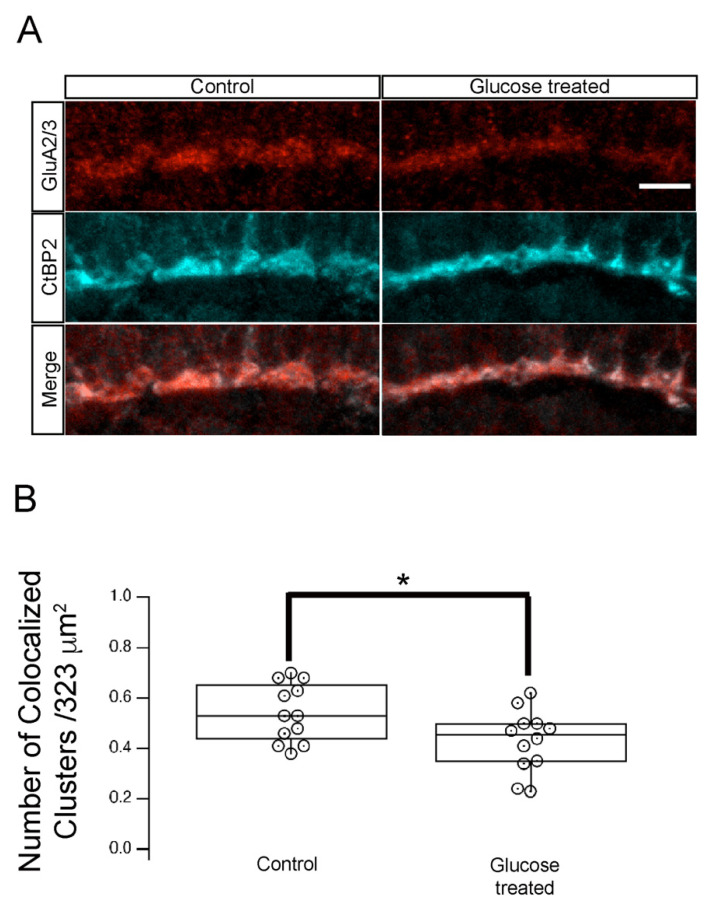
Hyperglycemia altered the synapse between photoreceptor and GluR2/3-containing neurons in the retinal INL. (**A**) Transverse retinal sections from 5 dpf control (**left panels**) and hyperglycemic (**right panels**) larvae were immunostained with fluorescently labeled antibodies specific for CtBP2 (red, **top**) or GluR2/3 (cyan, **middle**); overlays of CtBP2 and GluR2/3 signals are shown (merge; **bottom**). Maximal intensity projections are shown. Scale bar, 5 µm. We observed co-localization of ribeye/CtBP2 and GluR2/3 proteins in the OPL. (**B**) Quantitative analyses of IHC for CtBP2 and GluR2/3 in the OPL shown by box-and-whisker plots that indicate the number of colocalized clusters from control and hyperglycemic larvae. Scale bar, 5µm. Boxes indicate interquartile ranges, while whiskers indicate range of maximal and minimal values. Data are presented as mean values ± SEM. * *p* < 0.05. Abbreviations used: CtBP2, C-terminal binding protein 2; dpf, days post-fertilization; GluR2/3, glutamate receptor 2/3; INL, inner nuclear layer; OPL, outer plexiform layer.

The glutamate receptor proteins (GluR2/3) are expressed in postsynaptic horizontal cell processes, dendrites of OFF bipolar cells, and in some bipolar cells, where they invaginate into rod spherules [30]. Therefore, these proteins serve as markers of the synapse between bipolar cells and photoreceptors. To determine whether the apparent defects in synaptic maturation we observed in hyperglycemic larvae resulted from altered photoreceptor formation or maintenance, we double labeled retinal sections from 5 dpf control or hyperglycemic larvae with fluorescently labeled antibodies specific for CtBP2 or GluR2/3. We detected band-like clusters of GluR2/3 immunoreactivity in the OPL of control larvae that largely overlapped the area of CtBP2 labeling (Figure 4A). Quantitative analysis showed that, in control larvae, 98.5  ±  0.9% (mean  ±  S.E.M) of GluR2/3 and CtBP2 cluster colocalized when we sampled 12 image volumes of mean volume 5,998 μm^3^ from 32 larvae (Figure 4B). In hyperglycemic larvae, we observed a similar cluster pattern of CtBP2 and GluR2/3 staining (Figure 4A) and a similar number of clusters of the two proteins (Figure 4B), but their overlap was significantly reduced to 78.5  ±  0.9% (mean  ±  S.E.M) colocalized GluR2/3 and CtBP2 cluster. These data suggest that the number of OPL ribbon synapses established between photoreceptors and GluR2/3-expressing OFF bipolar cells was impaired in hyperglycemic zebrafish larvae.

**Figure 5:**
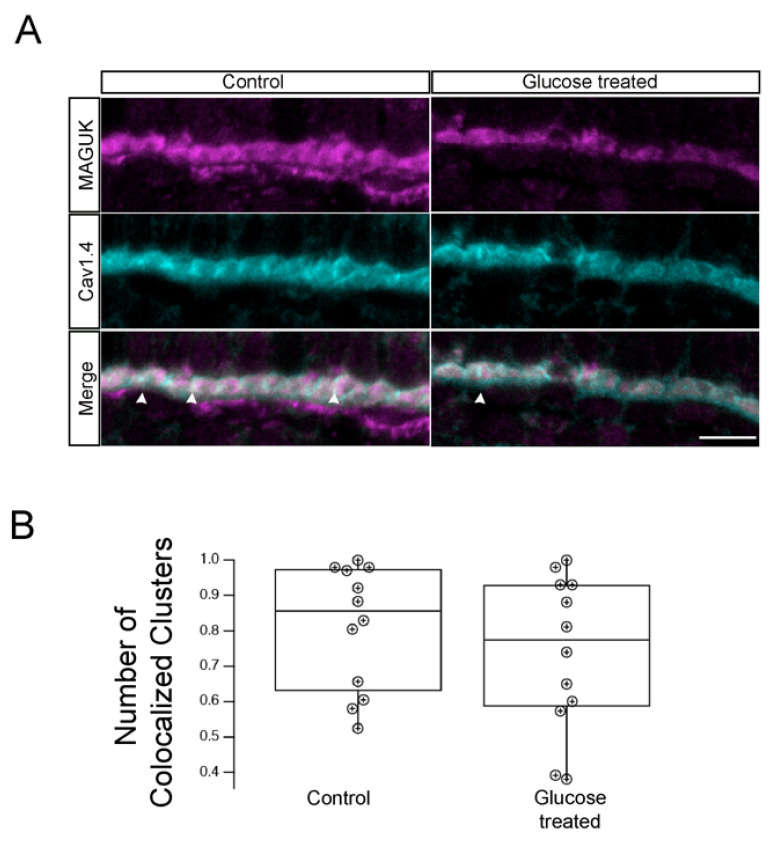
Hyperglycemic zebrafish larvae retained photoreceptor ribbon synapses with postsynaptic densities (PSDs) in INL dendrites. (**A**)Transverse retinal cross-sections from 5 dpf control (**left panels**) or hyperglycemic (**right panels**) larvae were double immunostained with fluorescently labeled antibodies specific for the postsynaptic marker MAGUK (magenta, **top**) or Cav1.4/cacna1f (cyan, **middle**); overlays of MAGUK and Cav1.4 labeling are shown (merge, **bottom**). Maximal intensity projections are shown; arrowheads indicate potential areas of colocalization. Scale bar, 5 µm. (**B**) Quantitative analyses of IHC for MAGUK and Cav1,4 colocalization are indicted by box-and-whisker plots. Boxes indicate interquartile ranges, while whiskers indicate range of maximal and minimal values. Scale bar, 5 µm. Abbreviations used: Cav1.4/cacna1f, voltage-dependent calcium channel; Dpf, days post-fertilization; INL, inner nuclear layer; MAGUK, membrane-associated guanylate kinase; PSD, postsynaptic densities; SEM, standard error of the mean.

To determine whether this reduction in the number of OPL synapses was a general phenomenon in hyperglycemic larvae, we examined the localization of Cav1.4 and another postsynaptic marker, the PSD-95 family membrane-associated guanylate kinase MAGUK, which assembles and regulates the postsynaptic densities of the excitatory synapse [31,32]. We co-immunolabeled 5 dpf control and hyperglycemic larvae with antibodies specific for presynaptic marker Ca_v_1.4 or postsynaptic marker MAGUK and performed IHC analysis as before. We observed labeling of MAGUK that appeared to partially overlap that of Ca_v_1.4 in the retinal OPL in a manner similar to that observed with CtBP2 and Ca_v_1.4/cacna1f (Figure 5). The quantitative analysis showed that 81.1 ± 0.5% and 73.5  ±  0.6% (mean  ±  S.E.M) MAGUK and Cav1.4 cluster colocalized in control and hyperglycemia at 120 hpf, although this difference was not statistically significant (Figure 5B).

**Figure 6:**
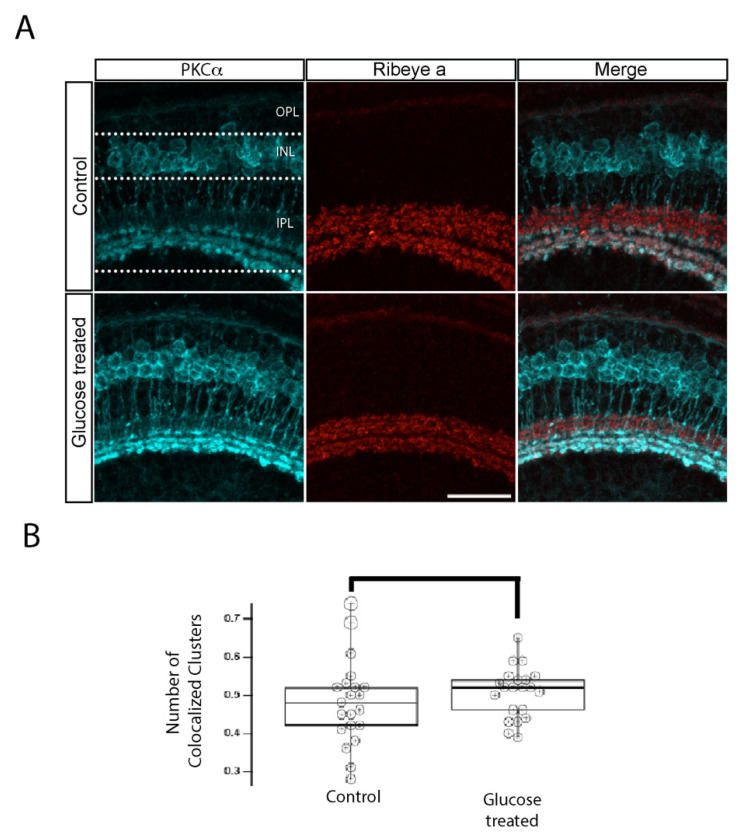
IPL ribbon synapses of bipolar cells are preserved in hyperglycemic larvae. (**A**) Transverse retinal sections from 5 dpf control (**top**) and hyperglycemic (**bottom**) larvae were double immunostained with fluorescently labeled antibodies specific for the rod bipolar cell marker PKCα (cyan; **left panels**) or *ribeye a* (red; **middle panels**); also shown is the overlay of PKCα and *ribeye a* labeling (merge; **right panels**). Maximal intensity projections are shown, and the relative positions of the OPL, INL, and IPL are indicated. Scale bar, 5 µm. The expression pattern of PKCα-labeled rod bipolar cell neurons, synaptic ribbons in the IPL layer, and the number of ribbon synapses in rod bipolar cells are normal in 5 dpf hyperglycemic larvae. (**B**) Quantitative analyses of IHC for colocalization of PKCα and *ribeye a* are indicated by box-and-whisker plots. Boxes indicate median values, and whiskers indicate 5th–95th percentile values of pooled data from 56 larvae. The data represent the total *ribeye a* labeling in PKCα-labeled terminals (*p* > 0.05). Abbreviations used: Dpf, days post-fertilization; PKCα, protein kinase C alpha; SEM, standard error of the mean.

In light of our observation that the maturation of *ribeye b* protein in the OPL synapse was impaired in 5 dpf hyperglycemic larvae (Figure 2), we hypothesized that expression of the *ribeye a* protein in the IPL would also be altered in these larvae. *Ribeye a* is required for the complete development and survival of bipolar cells [14]. To reveal the predicted changes in IPL ribbon synapses, we double immunostained control and hyperglycemic larvae with fluorescently labeled antibodies specific for *ribeye a* and for the rod bipolar cell marker PKCα. As expected, the *ribeye a* antibodies strongly labeled numerous clusters in the IPL, but the signal was faint in the OPL (Figure 6A), consistent with previous reports of differential expression of *ribeye a* in the retina [14]. The number of clusters of *ribeye a* staining in the IPL was similar between the two groups. The sizes of the IPL clusters varied, with some appearing larger in the retinas of hyperglycemic larvae, but these differences were not statistically significant (Figure 6A). Nevertheless, this trend was consistent with the levels of *ribeye a* mRNA after 48 h (Figure 1C). The retinas of both control and hyperglycemic groups were robustly stained with anti-PKCα antibodies (Figure 6A). At 5 dpf, the soma of rod bipolar cells in both groups exhibited a hexagonal shape (Figure 6A) rather than the characteristic pear shape expected of adult rod bipolar cells [33]. In hyperglycemic larvae, the rod bipolar cells appeared to be shorter and their termini larger and less organized than those in control larvae (Figure 6A), but these differences were not consistent between animals. As expected, in 5 dpf larvae, the clusters of *ribeye a* colocalized with rod bipolar cell terminals primarily located in sublamina *b* of the IPL (Figure 6A). These findings suggest that changes in IPL ribbon synapses in hyperglycemic larvae were marginal and may not reflect the visual deficits previously reported in this model.

If these visual deficits resulted from altered photoreceptor ribbon maturation, it should affect the development of synaptic vesicles when presynaptic defects in hyperglycemic larvae are first apparent. At 2.5 dpf, the transmitter-filled synaptic vesicles at the ventronasal patch fuse to form the presynaptic terminal membrane [15,34], and any changes to the pattern of synaptic vesicle protein expression can affect the signal transmission from primary neurons. The most abundant synaptic vesicle protein, synaptophysin [35], is expressed in all retinal vesicular synapses [36,37,38]. The synaptic functions of synaptophysin include synapse protein biogenesis, synapse formation, and cellular trafficking processes of exocytosis and endocytosis, eventually leading to neurotransmitter release [39,40,41,42,43,44,45,46,47,48,49].

**Figure 7:**
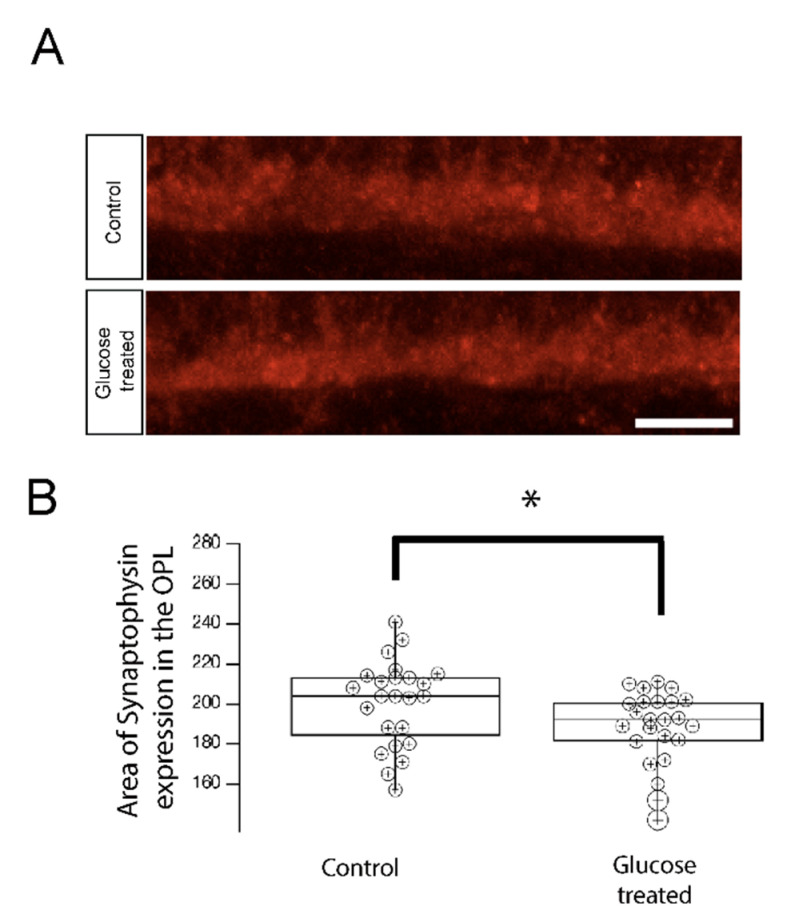
Synaptophysin accumulation in the OPL is reduced in 5 dpf hyperglycemic larvae. (**A**) Transverse retinal sections from 5 dpf control and hypoglycemic larvae were immunostained with fluorescently labeled antibodies specific for the synaptic vesicle marker synaptophysin. Maximal intensity projections are shown. Scale bar, 5 µm. Synaptophysin accumulation was reduced in hyperglycemic larvae relative to that in control larvae. (**B**) Quantitative analyses of IHC for synaptophysin accumulation in control and hyperglycemic larvae are indicated by box-and-whisker plots. Boxes indicate median value ranges, while whiskers indicate 5th–95th percentile values. Data represent the average value of the area of expression in 6–9 animals obtained from 3–5 independent experiments we sampled per condition, shown as individual data points. Scale bar, 5 µm. * *p* < 0.05; two-tailed *t*-test. Abbreviations used: dpf, days post-fertilization; SEM, standard error of the mean.

Therefore, we examined the expression pattern of synaptophysin in 5 dpf hyperglycemic larvae. Although synaptophysin is known to be present in both the IPL and OPL, the lack of statistically significant differences in its levels in IPL synapses led us to focus on its expression in the OPL. We stained control and hyperglycemic larvae with fluorescently labeled antibodies specific for synaptophysin and performed IHC as before (Figure 7A). We quantified the synaptophysin-positive area within the OPL and normalized it to 100 μm of retinal length (Figure 7B). We observed a statistically significant reduction in the size of the synaptophysin-positive area of the OPL in hyperglycemic larvae, relative to control larvae (Figure 7), suggesting that the accumulation of synaptic vesicles may be reduced. These findings provide additional evidence that the visual defect in hyperglycemic zebrafish larvae may be related to the impairment of photoreceptor ribbon maturation.

## 3. Discussion

Diabetic retinopathy is a prevalent complication of DM that can result in progressive blindness in adults. Gestational DM is associated with changes in retinal development and may extend to adulthood [4,7]. Changes in diabetic retinopathy observed in humans and animal models include altered retinal morphology, retinal thinning, loss of neuronal cells and Müller glial cells, increased accumulation of macrophages, increased retinal oxidative stress, and abnormal visual behavior in both adults and offspring [4,7,8]. However, the synaptic basis for such visual deficits has never been examined until recently. A study in which glucose overload altered the genes encoding presynaptic and postsynaptic proteins identified synaptotagmins 2 and 4 as novel glucose-responsive transcription factors in the cAMP response element-binding protein (CREB) family that are involved in impairment of synaptic function during hyperglycemia [50]. These findings prompted us to examine whether hyperglycemia affects the development of retinal synapses.

Zebrafish provide a valuable animal model for type 2 DM when the animals are immersed in a glucose solution. We previously demonstrated that excess glucose in the medium will passively diffuse into the embryos [7], resulting in increased blood glucose levels, degeneration of the retina, and visual impairment in hyperglycemic larvae and adults. [7,8]. Glucose also accumulates in the developing retina of glucose-exposed embryos, validating this model for diabetic retinopathy.

During development, the establishment of correct synaptic connectivity is crucial for normal sensory and motor function. In early zebrafish development at 48 hpf, the retinal ganglion cell layer has already formed adjacent to the lens and distinguishable from the rest of the retina, while the remaining neuronal cell layers are not fully differentiated until after 60 hpf [51]. At 5 dpf, hyperglycemic larvae exhibit visual impairment characterized by decreased numbers of photoreceptors, stunted outer retinal segments, and loss of horizontal and ganglion cells [8]. These data raised questions about the synaptic integrity of the retinal synapses in these animals. Ours is the first study to examine the expression of synaptic proteins specifically at ribbon synapses in the hyperglycemic zebrafish model.

We found that in the retina of both control and hyperglycemic larvae at 120 hpf, the photoreceptor synaptic ribbons visualized by immunostaining with antibodies specific for CtBP2 exhibited the characteristic horseshoe shape but that in hyperglycemic larvae, the synaptic ribbons varied in size, relative to those in control larvae, and resembled the immature stages of synaptic ribbons [26]. Interestingly, we found that accumulation of the voltage-gated calcium channel Ca_v_1.4/cacna1f formed a pattern similar to that of CtBP2. This was not surprising in light of previous reports that Ca_v_1.4/cacna1f provides a structural scaffold for interactions with ribbon proteins in the photoreceptor ribbon synapse [29,52] and that the loss of *ribeye* protein affects the abundance or localization of L-type calcium channels in hair cell ribbon synapses [53,54]. A longer duration of hyperglycemia would help us determine whether the small synaptic ribbon substructures (synaptic bodies) we observed could develop into mature synaptic ribbons given enough time, in which case the apparently immature ribbons we detected at 5 dpf would be expected to result from a developmental delay. Alternatively, if these synaptic body structures were to remain with the same size at later developmental stages, they might be expected to result from impairment in the accumulation of the *ribeye* and Ca_v_1.4/cacna1f proteins. While our RT-qPCR results showing a decrease in *ribeye b* transcription fit with the latter hypothesis, the increase in Ca_v_1.4/cacna1f transcription that we observed at 50 hpf does not. One possible explanation for this apparent discrepancy in the results obtained for Ca_v_1.4/cacna1f with RT-qPCR and IHC could be that the RT-qPCR analysis was performed at 48 hpf, while the IHC was performed at 120 hpf. Considering that Type 2 DM is a protein misfolding disorder [55], another possible explanation could be that the increase in Cav1.4/cacna1f transcription may have resulted in misfolding of the protein product, such that it could have been misassembled, resulting in accumulation of immature synaptic ribbons or that it could have been rendered undetectable by inaccessibility of the epitope in the misfolded protein. Of note, our immunostaining of CtBP2 and Cav1.4/cacna1f proteins at 120 hpf demonstrated parallel changes that are consistent with the idea that synaptic ribbon formation may also be regulated by calcium influx via L-VDCCs. [26]. For example, the accumulation of the matured synaptic ribbon and Cav1.4/cacna1f in hyperglycemic zebrafish decreased by 1.3- and 1.6-fold, respectively, whereas the accumulation of Ca_v_1.4/cacna1f in both elongated and spherical-shaped immature synaptic ribbons in hyperglycemic zebrafish increased by 1.6- and 2.1-fold (elongated ribbons) and by 1.5- and 2.7-fold (spherical ribbons). A future study to compare the loss of specific photoreceptors and the development of mature OPL might reveal whether the delay/loss of OPL synapse development precedes the loss of photoreceptors or vice versa.

Another remarkable finding was the decrease in the number of OPL synapses we detected in hyperglycemic larvae relative to control larvae. Co-immunolabeling of retinal sections with antibodies specific for the postsynaptic cell marker GluR2/3 and presynaptic marker CtBP2 exhibited a decreased overlap between the two in hyperglycemic larvae. Since GluR2/3 accumulates in postsynaptic horizontal cell processes and dendrites of OFF bipolar cells [30], and the number of horizontal cells was reduced in 5 dpf larvae [7,8], we might predict a reduction in the amount of GluR2/3 detected at this time point. However, rather than a significant reduction in the number of GluR2/3 clusters, we observed a decrease in the overlap between GluR2/3 and CtBP2, suggesting that the remaining horizontal cells may have been remodeled to form ectopic synapses, as shown previously [56]. One possible explanation for this is that such extended horizontal process synapses may have formed but that we were unable to detect them because they failed to overlap well with presynaptic photoreceptor synaptic ribbons. It would be interesting in the future to determine whether ectopic synapse formation occurs in hyperglycemic zebrafish larvae using immunoreactivity to calbindin, which serves as a marker for horizontal cells in the mouse retina [57]. RT-qPCR analysis at 120 hpf demonstrated a reduction in *ribeye b* and GluR2/3 transcription, lending support to the decrease in protein overlap in the OPL synapse we detected in hyperglycemic larvae. However, changes in OPL may not be a general phenomenon, at least at 120 hpf, because specific immunolabeling of Ca_v_1.4/cacna1f and MAGUK showed no change in the overlap between these proteins in hyperglycemic larvae relative to control larvae.

Surprisingly, we did not detect any differences in IPL ribbon synapses in hyperglycemic larvae. The differences between control and hyperglycemic larvae in the size and numbers of the ribbons were not statistically significant at 5 dpf, and we detected no differences in the number of rod bipolar cells by PKCα labeling in the presence or absence of hyperglycemia. Some rod bipolar cell terminals appeared to be larger or improperly arranged in the lamina in hyperglycemic larvae, and some of the dendrites were shorter; yet, these differences between hyperglycemic and control larvae were not statistically significant at 5 dpf. It would be interesting to repeat this analysis at a later developmental stage to look for any significant changes in the accumulation of *ribeye a* protein or in the number or morphologies of rod bipolar cells. Finally, we found that accumulation of the synaptic vesicle protein synaptophysin in the OPL was reduced in a manner that was consistent with the delay we observed in maturation of the OPL synapse. Of note, the changes we found in OPL synapse maturation could partly be due to the reduced number of photoreceptors reported in previous hyperglycemic zebrafish studies [8]. The decreased number of matured photoreceptor synaptic ribbons, the reduced accumulation of Ca_v_1.4/cacna1f and synaptophysin, and the decreased detection of the OPL synapse in hyperglycemic zebrafish larvae may explain the visual deficit previously reported in this animal model.

## 4. Materials and Methods

### 4.1. Zebrafish Maintenance and Induction of Hyperglycemia

The University of Tennessee Health Science Center (UTHSC) and University of Mississippi Medical Center (UMMC) Institutional Animal Care and Use Committees (IACUC) approved our protocols for zebrafish husbandry and for the experiments described in this manuscript, and our use of animals in vision research complied with IACUC guidelines. Wild-type (WT) zebrafish of the wild India Kolkata (WIK strain) were obtained from the Zebrafish International Resource Center (ZIRC; Eugene, OR, USA). Fish were propagated by natural mating and maintained on a controlled cycle of 12 h of light and 12 h of darkness at 28.5 °C in the system water. The onset of fertilization was approximately 9:30 AM (30 min after lights were turned on). WT embryos were obtained from and raised in embryonic media (E3) under standard conditions in 12 h light: 12 h darkness at 27.5 to 28.5 °C, and their developmental stages were monitored according to established protocols (staging).

To generate hyperglycemic embryos and/or larvae, 50 zebrafish embryos were mock treated in E3 medium lacking glucose or subjected to pulsatile high glucose (4%) exposure, as described [7]. Starting at 24 h post-fertilization (hpf), embryos were housed for 24 h in E3 medium that contained 4% glucose, then alternated every 24 h between E3 medium and E3 medium containing 4% glucose for a total of five days, with the second (last) induction occurring 71–96 hpf. At 5 d post-fertilization (dpf), the embryos were humanely euthanized by rapid chilling, transferred to a 50 mL tube containing E3 medium at 2~4 °C, and placed in an ice bath for at least 20 min. After visually confirming the absence of a heartbeat for a period of approximately five minutes under a stereoscope (Olympus SZX16 Wide Zoom Versatile Stereo/fluorescent Microscope, tilting trinocular observation, Shinjuku, Tokyo, Japan), the embryos were fixed overnight at 4 °C with freshly prepared 4% paraformaldehyde (PFA; Electron Microscopy Sciences, Hatfield, PA, USA) in a glass vial, as described previously for carboxy-terminal binding protein 2 (CtBP2) [58]. Please note that the sexes of the larvae were not determined at 5 dpf.

### 4.2. Immunofluorescence of the Retina

To perform immunohistochemistry (IHC), fixed larvae were washed extensively in phosphate-buffered saline (PBS), cryoprotected with an increasing concentration of sucrose in PBS (10%, 20%, and finally 30% sucrose) until equilibrated, embedded in optimal cutting temperature (OCT) compound (Fisher Healthcare, Houston, TX, USA) in a Tissue-Tek Cryomold (Fisher Scientific, Pittsburg, PA), snap frozen, and stored at −80 °C. A Leica CM1850 cryostat (Leica Microsystems, Bannockburn, IL, USA) with a chamber temperature of −12 to −15 °C was used to cut 15 μm coronal sections, which were transferred to Superfrost™ Plus microscope slides (Fisher Scientific, Pittsburg, PA, USA) at a density of three sections per slide, and stored at −20 to −80 °C.

Microscope slides that contained good intact retinal cryosections were selected from each group and left to dry at room temperature for 2 h. A hydrophobic barrier was applied generously around the sections with a PAP pen (Electron Microscopy Sciences, Hatfield, PA, USA) and allowed to dry for 15 min. Subsequently, the slides were washed three times with 1X PBS, permeabilized, and blocked with a solution containing 0.3% Triton-X-100 and 5% donkey serum in PBS for 2 h at room temperature (RT), as described previously for zebrafish larvae [58]. Primary antibodies (Table 1) were added to fresh blocking solution and applied to the slides, which were incubated at room temperature overnight. After three washes, species-specific secondary antibodies were added for 1 h at RT. Cell soma were labeled with 300 nM DAPI (diluted from a 14.3 mM stock solution) for 5 m. Slides were washed (once in PBS-Tween, three times in PBS, and once in distilled water), Prolong Diamond Antifade Mountant (ThermoFisher Scientific, Waltham, MA, USA) was applied to each slide, and coverslips were added.

### 4.3. Data Acquisition and Image Analysis

Images were acquired using an Olympus FV-3000 laser-scanning confocal system (Olympus, Shinjuku, Tokyo, Japan) connected to an Olympus IX-83 inverted microscope, controlled by Olympus FV31S-SW software (Version 2.3.1.163) with a Galvano scanner and a 60 X silicon (NA, 1.3) objective, using the following settings: 1~7 times software zoom, z-step size of 0.45 μm, and sequential line scan averaged 3 times for scan size of 256 × 256 pixels at 10.0 μs/pixel. Images were analyzed using ImageJ (imagej.nih.gov, accessed on 1 September 2021) and Imaris 9.9 (Bitplane, Zurich, Switzerland) software. The acquisition parameters (laser power, PMT gain, scan speed, optical zoom, offset, step size, and pinhole diameter) were kept constant for each experimental dataset. Imaging and analysis were performed on the central retina across the group.

### 4.4. Analysis of Synaptic Proteins by IHC

For quantitative analyses of labeled structures in 5 dpf larvae, 15 μm thick confocal Z-stack images were collected (6–9 zebrafish for each group from 3–5 independent experiments). Mature synaptic ribbons were identified visually by their characteristic horseshoe/arch shape and counted manually using Fiji/ImageJ software (imagej.nih.gov, and pinhole diameter). Box-and-whisker plots were generated in Igor Pro 8 software (Wavemetrics, Lake Oswego, OR, USA). Spherical and ellipsoid shapes of synaptic ribbons were measured as described previously [26]. Briefly, the length of the elongated structures was measured in three segments along the ribbon, the length-to-width ratios were calculated, and structures were counted and categorized as spherical (ratio < 1) or ellipsoid (1 to 2.5) or elongated (ratio > 2.5). Image acquisition and quantitative analyses were performed by different researchers in a blinded fashion to reduce the possibility of bias.

### 4.5. Analysis of Ribbon Synapses

Colocalization between two proteins was obtained from the surface module in Imaris software (Bitplane, Zurich, Switzerland). Images were deconvoluted, and an Imaris median filter was applied to both channels to improve the quality of the image and reduce background noise. The surface rendering module was used to obtain a 3D structure that included immunolabeling of both presynaptic and postsynaptic marker proteins [64,65], and the colocalization percentage was calculated by using a “surface to surface distance” filter with a value set to less than zero.

### 4.6. Cell Counting

The oval shape of the ONL cell nucleus or the round shapes of INL or GCL cells were considered to be a neuron in the respective nuclear layer. Digital images corresponding to different retinal regions were overlaid with random boxes 100 × 100 mm in size. The number of cells in the ONL was manually counted using Fiji/ImageJ software, while the cells in the INL and GCL were counted using the surface module in Imaris software. Images were deconvolved, the median filter was applied, and the 100 × 100 μm^2^ regions of interest were defined as described above.

### 4.7. Glucose Measurement

Embryos were dissected to separate the retina from the rest of the body, homogenized in sodium phosphate buffer, pH 7.2, and the glucose levels in the retina and in the rest of the body were measured using a glucose calorimetric assay (Cayman, Ann Arbor MI, USA), according to the manufacturer’s protocol.

### 4.8. Whole-Mount In Situ Hybridization (WISH)

Embryos were fixed in 4%PFA-PBS overnight at 4 °C before being transferred to and stored in 100% methanol at –20 °C. Whole-mount in situ hybridization was performed using digoxigenin-labeled riboprobes, as described [66]. To generate the probe, the *ribeye a* gene was amplified by RT-PCR and cloned upstream of the bacteriophage SP6 promoter in the pCRII TOPO dual promoter vector (Invitrogen, Waltham MA, USA). The resulting plasmid was linearized with the restriction endonuclease NotI and used as a template for in vitro synthesis of antisense probe RNA with SP6 RNA polymerase in the presence of digoxigenin-labeled UTP.

### 4.9. Quantitative RT-PCR (RT-qPCR)

Embryos were snap frozen in liquid nitrogen, total mRNA was extracted using the Direct-zol RNA MiniPrep kit (ZYMO research, Irvine, CA, USA), and first-strand complementary DNA (cDNA) was generated using the iSrcipt reverse transcription mix (Bio-Rad Hercules, CA, USA). Quantitative PCR products were generated using the DreamTaq Green PCR mix (ThermoFisher scientific, Waltham, MA, USA). The primers used for qPCR allowed amplification of *ribeye a*, *ribeye b*, GluR2/3, Cav1.4, Cav1.3, and 18S ribosomal RNA (rRNA) for normalization are listed in Table 2.

### 4.10. Statistical Analysis

Statistical analyses were performed using Microsoft Excel (Version 16.59) and Igor Pro 8 (Wavemetrics, Lake Oswego, OR, USA) software. The differences in RNA and protein levels and the colocalization of synaptic proteins in control and hyperglycemic larvae were analyzed using unpaired two-tailed *t*-tests.

## Figures and Tables

**Table 1:** Antibodies used in this study.

Antigen	Antiserum	Host	Dilution	Source (Number)	Marker for	Reference
PKC	Monoclonal anti-PKC	Mouse	1:250	Santa Cruz (sc-17769)	Rod-type bipolar cells	[59]
Ribeye	Polyclonal anti-ribeye	Rabbit	1:1000	Zenisek lab (s4561-2)	Synaptic ribbons (IPL)	[60]
CtBP	Monoclonal anti-CtBP (b-3)	Mouse	1:100	Santa Cruz (sc-55502)	Synaptic ribbons (OPL and IPL)	[24]
Ca_v_1.4	Polyclonal anti-Ca2+ channel L-type alpha-1F	Rabbit	1:500	Synaptic Systems (365-003)	OPL photoreceptor terminals	[28]
Synaptophysin	Polyclonal anti-synaptophysin	Rabbit	1:250	Alomone lab (ANR-013)	Synaptic vesicles	[61]
Pan-MAGUK	Monoclonal pan-MAGUK, clone K28/86	Mouse	1:250	Millipore Sigma (MABN72MI)	Postsynaptic excitatory glutamatergic synapses	[62]
GluR2/3	Polyclonal anti-GluR2/3	Rabbit	1:250	Millipore Sigma (07-598)	OFF bipolar and horizontal cell dendrites	[63]

**Table 2:** Primers used for RT-qPCR analysis.

	Forward Primer	Reverse Primer
*Ribeye a*	CTATACCGTACCAATGGAGCTAATG	GCATCTCCACAGTACAGTCTCTC
*Ribeye b*	CAGAGCGGCTCCGAAGACGTTTTCCGGC	ACAGAAGGCAACGGTTGCCAGATC
Gria2a/GluR2a	GAGCAGGGGCTTCTGGATAAAT	TCTGTGAACGTCATCCCACTA
Ca_v_1.3a	ACAGGAACCAGTACCTGCAGA	CCATTTCGTCAATGGTCATCT
Ca_v_1.4a	CCCCTAGAAGCACGCCTATG	CCACTTGCTGGGTAAGGGAG
18S rRNA	TCGCTAGTTGGCATCGTTTATG	CGGAGGTTCGAAGACGATCA

## Data Availability

Available upon request.

## References

[1] Tang J., Kern T.S. (2011). Inflammation in diabetic retinopathy. Prog. Retin. Eye Res..

[2] El-Remessy A.B., Al-Shabrawey M., Khalifa Y., Tsai N.T., Caldwell R.B., Liou G.I. (2006). Neuroprotective and blood-retinal barrier-preserving effects of cannabidiol in experimental diabetes. Am. J. Pathol..

[3] Xie Z., Wei M., Morgan T.E., Fabrizio P., Han D., Finch C.E., Longo V.D. (2002). Peroxynitrite mediates neurotoxicity of amyloid beta-peptide1-42- and lipopolysaccharide-activated microglia. J. Neurosci..

[4] Tariq Y.M., Samarawickrama C., Li H., Huynh S.C., Burlutsky G., Mitchell P. (2010). Retinal thickness in the offspring of diabetic pregnancies. Am. J. Ophthalmol..

[5] Chhetri J., Jacobson G., Gueven N. (2014). Zebrafish—On the move towards ophthalmological research. Eye.

[6] Howe K., Clark M.D., Torroja C.F., Torrance J., Berthelot C., Muffato M., Collins J.E., Humphray S., McLaren K., Matthews L. (2013). The zebrafish reference genome sequence and its relationship to the human genome. Nature.

[7] Singh A., Castillo H.A., Brown J., Kaslin J., Dwyer K.M., Gibert Y. (2019). High glucose levels affect retinal patterning during zebrafish embryogenesis. Sci. Rep..

[8] Titialii-Torres K.F., Morris A.C. (2022). Embryonic hyperglycemia perturbs the development of specific retinal cell types, including photoreceptors. J. Cell Sci..

[9] Larison K.D., Bremiller R. (1990). Early onset of phenotype and cell patterning in the embryonic zebrafish retina. Development.

[10] Fadool J.M. (2003). Development of a rod photoreceptor mosaic revealed in transgenic zebrafish. Dev. Biol..

[11] Matthews G., Fuchs P. (2010). The diverse roles of ribbon synapses in sensory neurotransmission. Nat. Rev. Neurosci..

[12] Moser T., Grabner C., Schmitz F. (2020). Sensory Processing at Ribbon Synapses in the Retina and the Cochlea. Physiol. Rev..

[13] Schmitz F. (2009). The making of synaptic ribbons: How they are built and what they do. Neuroscientist.

[14] Wan L., Almers W., Chen W. (2005). Two ribeye genes in teleosts: The role of Ribeye in ribbon formation and bipolar cell development. J. Neurosci..

[15] Biehlmaier O., Neuhauss S.C., Kohler K. (2003). Synaptic plasticity and functionality at the cone terminal of the developing zebrafish retina. J. Neurobiol..

[16] Hermes B., Reuss S., Vollrath L. (1992). Synaptic ribbons, spheres and intermediate structures in the developing rat retina. Int. J. Dev. Neurosci..

[17] Allwardt B.A., Lall A.B., Brockerhoff S.E., Dowling J.E. (2001). Synapse formation is arrested in retinal photoreceptors of the zebrafish nrc mutant. J. Neurosci..

[18] Schmitz F., Königstorfer A., Südhof T.C. (2000). RIBEYE, a component of synaptic ribbons: A protein’s journey through evolution provides insight into synaptic ribbon function. Neuron.

[19] Holzhausen L.C., Lewis A.A., Cheong K.K., Brockerhoff S.E. (2009). Differential role for synaptojanin 1 in rod and cone photoreceptors. J. Comp. Neurol..

[20] Trapani J.G., Obholzer N., Mo W., Brockerhoff S.E., Nicolson T. (2009). Synaptojanin1 is required for temporal fidelity of synaptic transmission in hair cells. PLoS Genet..

[21] Van Epps H.A., Hayashi M., Lucast L., Stearns G.W., Hurley J.B., De Camilli P., Brockerhoff S.E. (2004). The zebrafish nrc mutant reveals a role for the polyphosphoinositide phosphatase synaptojanin 1 in cone photoreceptor ribbon anchoring. J. Neurosci..

[22] Dick O., tom Dieck S., Altrock W.D., Ammermüller J., Weiler R., Garner C.C., Gundelfinger E.D., Brandstätter J.H. (2003). The presynaptic active zone protein bassoon is essential for photoreceptor ribbon synapse formation in the retina. Neuron.

[23] Hombrebueno J.R., Chen M., Penalva R.G., Xu H. (2014). Loss of synaptic connectivity, particularly in second order neurons is a key feature of diabetic retinal neuropathy in the Ins2Akita mouse. PLoS ONE.

[24] Graffe M., Zenisek D., Taraska J.W. (2015). A marginal band of microtubules transports and organizes mitochondria in retinal bipolar synaptic terminals. J. Gen. Physiol..

[25] Hoshi H., Sato F. (2018). The morphological characterization of orientation-biased displaced large-field ganglion cells in the central part of goldfish retina. J. Comp. Neurol..

[26] Liu X., Kerov V., Haeseleer F., Majumder A., Artemyev N., Baker S.A., Lee A. (2013). Dysregulation of Ca(v)1.4 channels disrupts the maturation of photoreceptor synaptic ribbons in congenital stationary night blindness type 2. Channels.

[27] Jia S., Muto A., Orisme W., Henson H.E., Parupalli C., Ju B., Baier H., Taylor M.R. (2014). Zebrafish Cacna1fa is required for cone photoreceptor function and synaptic ribbon formation. Hum. Mol. Genet..

[28] Ryl M., Urbasik A., Gierke K., Babai N., Joachimsthaler A., Feigenspan A., Frischknecht R., Stallwitz N., Fejtová A., Kremers J. (2021). Genetic disruption of bassoon in two mutant mouse lines causes divergent retinal phenotypes. FASEB J..

[29] Dembla E., Dembla M., Maxeiner S., Schmitz F. (2020). Synaptic ribbons foster active zone stability and illumination-dependent active zone enrichment of RIM2 and Ca_v_1.4 in photoreceptor synapses. Sci. Rep..

[30] Hack I., Frech M., Dick O., Peichl L., Brandstätter J.H. (2001). Heterogeneous distribution of AMPA glutamate receptor subunits at the photoreceptor synapses of rodent retina. Eur. J. Neurosci..

[31] Meyer M.P., Trimmer J.S., Gilthorpe J.D., Smith S.J. (2005). Characterization of zebrafish PSD-95 gene family members. J. Neurobiol..

[32] Boeckers T.M. (2006). The postsynaptic density. Cell Tissue Res..

[33] Heidelberger R., Matthews G. (1992). Calcium influx and calcium current in single synaptic terminals of goldfish retinal bipolar neurons. J. Physiol..

[34] Schmitt E.A., Dowling J.E. (1999). Early retinal development in the zebrafish, Danio rerio: Light and electron microscopic analyses. J. Comp. Neurol..

[35] Takamori S., Holt M., Stenius K., Lemke E.A., Grønborg M., Riedel D., Urlaub H., Schenck S., Brügger B., Ringler P. (2006). Molecular anatomy of a trafficking organelle. Cell.

[36] Dhingra N.K., Ramamohan Y., Raju T.R. (1997). Developmental expression of synaptophysin, synapsin I and syntaxin in the rat retina. Dev. Brain Res..

[37] Brandstätter J.H., Löhrke S., Morgans C.W., Wässle H. (1996). Distributions of two homologous synaptic vesicle proteins, synaptoporin and synaptophysin, in the mammalian retina. J. Comp. Neurol..

[38] Wiedenmann B., Franke W.W. (1985). Identification and localization of synaptophysin, an integral membrane glycoprotein of Mr 38,000 characteristic of presynaptic vesicles. Cell.

[39] Khvotchev M.V., Südhof T.C. (2004). Stimulus-dependent dynamic homo- and heteromultimerization of synaptobrevin/VAMP and synaptophysin. Biochemistry.

[40] Cameron P.L., Südhof T.C., Jahn R., De Camilli P. (1991). Colocalization of synaptophysin with transferrin receptors: Implications for synaptic vesicle biogenesis. J. Cell Biol..

[41] Eshkind L.G., Leube R.E. (1995). Mice lacking synaptophysin reproduce and form typical synaptic vesicles. Cell Tissue Res..

[42] Leube R.E., Wiedenmann B., Franke W.W. (1989). Topogenesis and sorting of synaptophysin: Synthesis of a synaptic vesicle protein from a gene transfected into nonneuroendocrine cells. Cell.

[43] Tarsa L., Goda Y. (2002). Synaptophysin regulates activity-dependent synapse formation in cultured hippocampal neurons. Proc. Natl. Acad. Sci. USA.

[44] Thiele C., Hannah M.J., Fahrenholz F., Huttner W.B. (2000). Cholesterol binds to synaptophysin and is required for biogenesis of synaptic vesicles. Nat. Cell Biol..

[45] Thomas L., Hartung K., Langosch D., Rehm H., Bamberg E., Franke W.W., Betz H. (1988). Identification of synaptophysin as a hexameric channel protein of the synaptic vesicle membrane. Science.

[46] Shrestha A., Vaithianathan T. (2022). Tracking the dynamics of single fused synaptic vesicle proteins from a single ribbon active zone in zebrafish retinal bipolar cells. STAR Protoc..

[47] Vaithianathan T., Henry D., Akmentin W., Matthews G. (2016). Nanoscale dynamics of synaptic vesicle trafficking and fusion at the presynaptic active zone. Elife.

[48] Bennett M.K., Calakos N., Scheller R.H. (1992). Syntaxin: A synaptic protein implicated in docking of synaptic vesicles at presynaptic active zones. Science.

[49] Greengard P., Valtorta F., Czernik A.J., Benfenati F. (1993). Synaptic vesicle phosphoproteins and regulation of synaptic function. Science.

[50] Ripoli C., Spinelli M., Natale F., Fusco S., Grassi C. (2020). Glucose Overload Inhibits Glutamatergic Synaptic Transmission: A Novel Role for CREB-Mediated Regulation of Synaptotagmins 2 and 4. Front. Cell Dev. Biol..

[51] Malicki J., Neuhauss S.C., Schier A.F., Solnica-Krezel L., Stemple D.L., Stainier D.Y., Abdelilah S., Zwartkruis F., Rangini Z., Driever W. (1996). Mutations affecting development of the zebrafish retina. Development.

[52] Maxeiner S., Luo F., Tan A., Schmitz F., Südhof T.C. (2016). How to make a synaptic ribbon: RIBEYE deletion abolishes ribbons in retinal synapses and disrupts neurotransmitter release. EMBO J..

[53] Sheets L., Trapani J.G., Mo W., Obholzer N., Nicolson T. (2011). Ribeye is required for presynaptic Ca_V_1.3a channel localization and afferent innervation of sensory hair cells. Development.

[54] Lv C., Stewart W.J., Akanyeti O., Frederick C., Zhu J., Santos-Sacchi J., Sheets L., Liao J.C., Zenisek D. (2016). Synaptic Ribbons Require Ribeye for Electron Density, Proper Synaptic Localization, and Recruitment of Calcium Channels. Cell Rep..

[55] Mukherjee A., Morales-Scheihing D., Butler P.C., Soto C. (2015). Type 2 diabetes as a protein misfolding disease. Trends Mol. Med..

[56] Sonntag S., Dedek K., Dorgau B., Schultz K., Schmidt K.F., Cimiotti K., Weiler R., Löwel S., Willecke K., Janssen-Bienhold U. (2012). Ablation of retinal horizontal cells from adult mice leads to rod degeneration and remodeling in the outer retina. J. Neurosci..

[57] Haverkamp S., Wässle H. (2000). Immunocytochemical analysis of the mouse retina. J. Comp. Neurol..

[58] Zanazzi G., Matthews G. (2010). Enrichment and differential targeting of complexins 3 and 4 in ribbon-containing sensory neurons during zebrafish development. Neural Dev..

[59] Wei X., Luo Y., Hyde D.R. (2006). Molecular cloning of three zebrafish lin7 genes and their expression patterns in the retina. Exp. Eye Res..

[60] Sheets L., Hagen M.W., Nicolson T. (2014). Characterization of Ribeye subunits in zebrafish hair cells reveals that exogenous Ribeye B-domain and CtBP1 localize to the basal ends of synaptic ribbons. PLoS ONE.

[61] Schmitt U., Tanimoto N., Seeliger M., Schaeffel F., Leube R.E. (2009). Detection of behavioral alterations and learning deficits in mice lacking synaptophysin. Neuroscience.

[62] Sebe J.Y., Cho S., Sheets L., Rutherford M.A., von Gersdorff H., Raible D.W. (2017). Ca^2+^-Permeable AMPARs Mediate Glutamatergic Transmission and Excitotoxic Damage at the Hair Cell Ribbon Synapse. J. Neurosci..

[63] Medalla M., Luebke J.I. (2015). Diversity of Glutamatergic Synaptic Strength in Lateral Prefrontal versus Primary Visual Cortices in the Rhesus Monkey. J. Neurosci..

[64] Fogarty M.J., Hammond L.A., Kanjhan R., Bellingham M.C., Noakes P.G. (2013). A method for the three-dimensional reconstruction of Neurobiotin-filled neurons and the location of their synaptic inputs. Front. Neural Circuits.

[65] Testen A., Kim R., Reissner K.J. (2020). High-Resolution Three-Dimensional Imaging of Individual Astrocytes Using Confocal Microscopy. Curr. Protoc. Neurosci..

[66] Fraher D., Ellis M.K., Morrison S., McGee S.L., Ward A.C., Walder K., Gibert Y. (2015). Lipid Abundance in Zebrafish Embryos Is Regulated by Complementary Actions of the Endocannabinoid System and Retinoic Acid Pathway. Endocrinology.

